# An Integrated Approach of Fuzzy Linguistic Preference Based AHP and Fuzzy COPRAS for Machine Tool Evaluation

**DOI:** 10.1371/journal.pone.0133599

**Published:** 2015-09-14

**Authors:** Huu-Tho Nguyen, Siti Zawiah Md Dawal, Yusoff Nukman, Hideki Aoyama, Keith Case

**Affiliations:** 1 Department of Mechanical Engineering, Faculty of Engineering, University of Malaya, 50603, Kuala Lumpur, Malaysia; 2 School of Integrated Design Engineering, Keio University, Tokyo, Japan; 3 Mechanical and Manufacturing Engineering, Loughborough University, Leicestershire, LE11 3TU, United Kingdom; Southwest University, CHINA

## Abstract

Globalization of business and competitiveness in manufacturing has forced companies to improve their manufacturing facilities to respond to market requirements. Machine tool evaluation involves an essential decision using imprecise and vague information, and plays a major role to improve the productivity and flexibility in manufacturing. The aim of this study is to present an integrated approach for decision-making in machine tool selection. This paper is focused on the integration of a consistent fuzzy AHP (Analytic Hierarchy Process) and a fuzzy COmplex PRoportional ASsessment (COPRAS) for multi-attribute decision-making in selecting the most suitable machine tool. In this method, the fuzzy linguistic reference relation is integrated into AHP to handle the imprecise and vague information, and to simplify the data collection for the pair-wise comparison matrix of the AHP which determines the weights of attributes. The output of the fuzzy AHP is imported into the fuzzy COPRAS method for ranking alternatives through the closeness coefficient. Presentation of the proposed model application is provided by a numerical example based on the collection of data by questionnaire and from the literature. The results highlight the integration of the improved fuzzy AHP and the fuzzy COPRAS as a precise tool and provide effective multi-attribute decision-making for evaluating the machine tool in the uncertain environment.

## Introduction

The globalization of business has required companies to be more productive and competitive. One of the methods to increase productivity is by improving the manufacturing facilities, such as introducing new production processes and equipment. The decision for procurement of new machines should be carefully made since inappropriate selection of machineries can negatively affect the overall performance of system operations in terms of productivity, precision, flexibility, adaptability and responsiveness [1]. This decision-making procedure involves the process of selecting the most appropriate solution among a set of numerous alternatives [2]. It is a time-consuming procedure, and achieving the optimal solution is difficult to be obtained [3] due to the lack of knowledge and experience, and proper understanding of technologies of the decision makers [4, 5].

The development of the production economy always requires companies to find a potential manufacturing solution to respond and satisfy the demand of customers. One of the important strategies to meet the optimal operational performance is applying production automation by implementation of flexible manufacturing systems (FMS) [1]. FMS can work both as a method for the efficient implementation of automated mass production operation and also for the flexibility of job shops where the simultaneous production of several part types is essential [6]. The typical FMS automatic batch manufacturing system would usually comprise of several computer numerical controlled (CNC) machine tools, workstations and a material handling system mechanically linked together and controlled by a computer-centered control system [7]. However, the investment cost for FMS is very high and is out of reach for most small and medium enterprises (SMEs). A smaller scale approach, with less number of machines, is the flexible manufacturing cells (FMCs), which requires lower investment costs but has reduced production rate and annual capacity. Machine tools form a critical component of both FMS and FMC, and their proper selection is an important task in equipment planning of FMCs [8].

Several techniques have been proposed for the decision-making process to evaluate the most suitable potential machines. Ayağ and Özdemir [9] have used a fuzzy AHP, approach by considering quantitative and qualitative attributes in the MCDM (Multi-Criteria Decision-Making) model. The fuzzy logic is utilized in solving the vague and imprecise information of the uncertain judgments from experts. The fuzzy AHP is used to determine the weights of criteria and the ranking of alternatives through the priority weights of alternatives. Finally, the Benefit/Cost (B/C) ratio analysis is implemented for each alternative, and the machine tool with the highest B/C ratio is selected. Taha and Rostam [1] have incorporated the use of Artificial Neural Network (ANN) in their development of a decision support system (DSS) based on multiple criteria for machine tool selection in flexible manufacturing cells using a fuzzy AHP and ANN. The ANN with feedback propagation is utilized to validate the results of the fuzzy AHP and to predict the ranking of potential alternatives. Önüt *et al*. [10] have described a hybrid fuzzy MCDM approach based on the integration of a fuzzy AHP and a fuzzy TOPSIS (Technique for Order Preference by Similarity to Ideal Solution) for evaluating and selecting vertical CNC machining centers. The priority weights of criteria were calculated through the fuzzy AHP to handle the qualitative criteria, and the results from the ranking of the alternatives are obtained by the fuzzy TOPSIS. Furthermore, Ayağ [11] has presented the integration of the AHP and simulation techniques for machine tool selection. Taha and Rostam [12] have presented a DSS for selection of the best machine in FMC using the hybrid method of the fuzzy AHP and PROMETHEE (Preference Ranking Organization METHod for Enrichment Evaluation) and Dağdeviren [13] has also proposed the integration of AHP and PROMETHEE. Durán and Aguilo [14] have used a fuzzy AHP approach for machine tool selection. Abdi [15] proposed the fuzzy AHP for MCDM in the equipment selection of reconfigurable machining systems. Ic *et al*.[16] have developed a machining center selection model based on the components of machines using AHP. Lin and Yang [17] also used the AHP for evaluation of machine selection. Ic and Yurdakul [18] have developed a DSS to select the most suitable machining center. This DSS involves the integration of the fuzzy AHP for calculating the priorities of criteria and fuzzy TOPSIS is employed for ranking the alternatives. Qi [19] has proposed a fuzzy MCDM approach based on the modified fuzzy AHP and grey theory for machine tool selection, including both qualitative and quantitative criteria, to determine the weights of criteria and the synthetic performance of each alternative through the Sugeno fuzzy integral. Lastly, Hasan Aghdaie *et al*. [20] have proposed the integration of SWARA (Step-wise weight assessment ratio analysis) and COPRAS-G methods for decision making in machine tool evaluation and selection. The three hybrid methods of SWARA-TOPSIS, SWARA-ELECTRE III and SWARA-VIKOR were described in solving decision-making problems [21]. Table 1 lists past research in machine selection process, showing the common MCDM method used in the respective studies.

**Table 1:** The previous research work on the approaches for machine tool evaluation.

Author	Year	Methodology
Ayağ and Özdemir [9]	2006	Fuzzy AHP
Taha and Rostam [1]	2011	Fuzzy AHP and ANN
Önüt *et al*. [10]	2008	Fuzzy AHP and Fuzzy TOPSIS
Ayağ [11]	2007	AHP and Simulation
Taha and Rostam [12]	2011	Fuzzy AHP and PROMETHEE
Myint and Tabucanon [22]	1994	AHP and GP, sensitivity analysis
Tabucanon *et al*. [23]	1994	AHP and Expert System
Yurdakul [24]	2004	AHP and ANP
Samvedi *et al*. [25]	2011	Fuzzy AHP and GRA
Durán and Aguilo [14]	2008	Fuzzy AHP
Dağdeviren [13]	2008	AHP and PROMETHEE
Paramasivam *et al*. [26]	2011	AHP and ANP
Ic *et al*. [16]	2012	AHP
İç and Yurdakul [18]	2009	Fuzzy AHP and Fuzzy TOPSIS
Lin and Yang [17]	1996	AHP
Abdi [15]	2009	Fuzzy AHP and sensitivity
Qi [19]	2010	Fuzzy AHP
Ayağ and Gürcan Özdemir [27]	2012	Fuzzy ANP and TOPSIS
Ayağ and Özdemir [28]	2011	Fuzzy ANP
Nguyen *et al*. [29]	2014	Fuzzy ANP and COPRAS-G
Chakraborty [30]	2011	MOORA
Özgen *et al*. [31]	2011	Modified DELPHI, AHP, PROMETHEE, Fuzzy sets
Tsai *et al*. [32]	2010	AHP
Yurdakul and Iç [33]	2009	Fuzzy TOPSIS
Balaji *et al*. [34]	2009	ELECTRE III
Sun *et al*. [35]	2008	AHP
Ertuğrul and Güneş [5]	2007	Fuzzy TOPSIS
Rao [36]	2006	digraph and matrix methods
Rao [37]	2007	GTMA, SAW, WPM, AHP, TOPSIS
Chtourou *et al*. [38]	2005	Expert System
Wang *et al*. [39]	2000	Fuzzy logic
Arslan *et al*. [4]	2004	Multi-criteria weighted average
Hasan Aghdaie *et al*. [20]	2013	SWARA and COPRAS-G

The review of past works has shown that fuzzy AHP is a suitable approach for multi-attribute decision-making process. The fuzzy AHP has been used to obtain reliable results in evaluating the alternatives and so is widely used in the uncertain environment. A significant advantage of the fuzzy AHP method is the capacity of generating the weights of attributes and the priorities of alternatives from the pair-wise comparison matrices of experts’ judgments. However, there are some disadvantages in the fuzzy AHP, particularly in collecting the judgments for the decision matrices since the process for data collection can be very time-consuming [5].

To overcome the limitations of the fuzzy AHP technique, this paper introduces the integration of a consistent fuzzy AHP and fuzzy COPRAS for machine tool selection. The determination of the Consistency Ratio (CR) is avoided when the fuzzy linguistic preference relation is employed to integrate into the AHP. The proposed method of machine tool selection is developed to be easily implemented.

## The Integration of the Consistent Fuzzy AHP and Fuzzy COPRAS for Machine Tool Selection

### The consistent fuzzy AHP

The AHP is a multi-criteria decision making technique that allows the decision-makers to structure the decision issues based on pairwise comparisons and expert’s judgments [40, 41]. The AHP presented by Saaty in 1980 (described in Ref. [42]) has become the most popular multi-criteria decision making method [43]. In the manufacturing environment, many industrial problems could not be solved because of incomplete or non-availability of information. Approximation approach, such as fuzzy logic can therefore be used to solve those uncertain problems. The fuzzy AHP combines the pair-wise comparison matrix of decision makers’ judgments and theory of fuzzy sets to handle the uncertainty problems. This method has become well-known for the multi-attribute decision-making (MADM) process, and the integration of fuzzy logic and AHP is a robust and flexible MADM tool for solving complex decision problems [44].

Existing fuzzy AHP uses the pair-wise comparison matrix with the collection of n(n-1)/2 comparisons. A questionnaire is normally used to get feedback from experts’ judgments. Hence, as the number of attribute increases, the pair-wise comparison questions and the complexity of the questionnaire increase. As the number of question in the survey increases, there is an increased possibility of respondents replying with inaccurate information. This can create inconsistencies in the results even though the consistency ratio may not be less than 0.1. These inconsistencies may require the experts to check and re-answer the questions, which is inefficient and a waste of time [42].

Fuzzy preference relation can be used to overcome this problem, and effective decisions in the practical decision-making process can be made [45–48, 49, 50]. Wang and Chen [51], Rezaei and Ortt [52] and Franco [46] proposed the integration of consistent fuzzy preference relations (CFPR) in the AHP approach to improve the consistency of fuzzy AHP. Using CFPR, the number of pair-wise comparisons are dramatically reduced from n(n-1) to (n-1) comparisons, and subsequently the remaining comparisons can be computed through the fuzzy preference relations. Thus, the process becomes more efficient, and decision makers take less effort to focus more on making the pair-wise comparisons of attributes [42]. For example, if there are ten attributes and five alternatives, there will be eleven pair-wise comparison matrices. In particular, one 10x10 pair-wise comparison matrix for attributes contains 10(10–1)/2 = 45 judgments and ten 5x5 pairwise comparison matrices contain 10*5(5–1)/2 = 100 judgments. Thus, the minimum number of judgments collected from experts must be 145 judgments. In addition, in evaluating alternatives, it is important that the consistency ratio (CR) must be less than 0.1. If the CR is greater than 0.1, then the judgments among the attributes and alternatives need to be re-evaluated. In contrast, using the improved consistent fuzzy AHP and fuzzy COPRAS, the number of pair-wise comparisons is only (10–1) = 9. Other similar approach using hybrid AHP concepts includes VIKOR (VIse Kriterijumska Optimizacija Kompromisno Resenje) [53], SAW (Simple Additive Weighting) [37], PROMETHEE (Preference Ranking Organization METHod for Enrich Evaluation) [12, 13], ELECTRE III (Elimination and Et Choice Translating Reality) [34], TOPSIS (Technique for Order Preference by Similarity to Ideal Solution) [5, 10, 14, 18, 31, 33, 54, 55] and ARAS (Additive Ratio Assessment) [56]. The integrated approach proposed in this paper, is actually practical and accurate in decision-making processes involving conflicting attributes which can cater for imprecise, uncertain information.

### Fuzzy COPRAS

The COPRAS (COmplex PRoportional ASsessment) introduced in 1996 by Zavadskas et al. [57], is a well-known MADM approach for evaluating and selecting the most appropriate alternative among a set of available potential alternatives. In this technique, the most suitable solution is determined based on the comparison between the direct and proportional ratio of the best solution and the ratio of the ideal-worst solution. It is constructed based on the attributes of alternatives to handle the complex real-world problems where the properties of attributes are conflicting [58]. However, the properties of the attributes and the expert’s judgments may contain uncertain and imprecise information. Thus, the classical multi-attribute decision-making approaches are insufficient to model the complex real-world problems. Thus, the fuzzy sets theory is the most suitable to be used for handling problems in uncertain environments. In this paper, the fuzzy sets are integrated into the COPRAS method in the form of fuzzy COPRAS [59]. The technique has been used by Chatterjee and Bose [58] for site selection of wind farms, by Fouladgar *et al*. [59] in evaluating the working strategies at a construction company and by Yazdani *et al*. [60] for risk analysis of critical infrastructures.

In this study, the fuzzy COPRAS would be applicable if the weights of the attributes and the ranking of the machine alternatives are given by fuzzy linguistic variables. These are addressed using the fuzzy numbers with input from experts’ judgments. The procedure of fuzzy COPRAS is described in Appendix A.2.

### The proposed model

**Fig 1:**
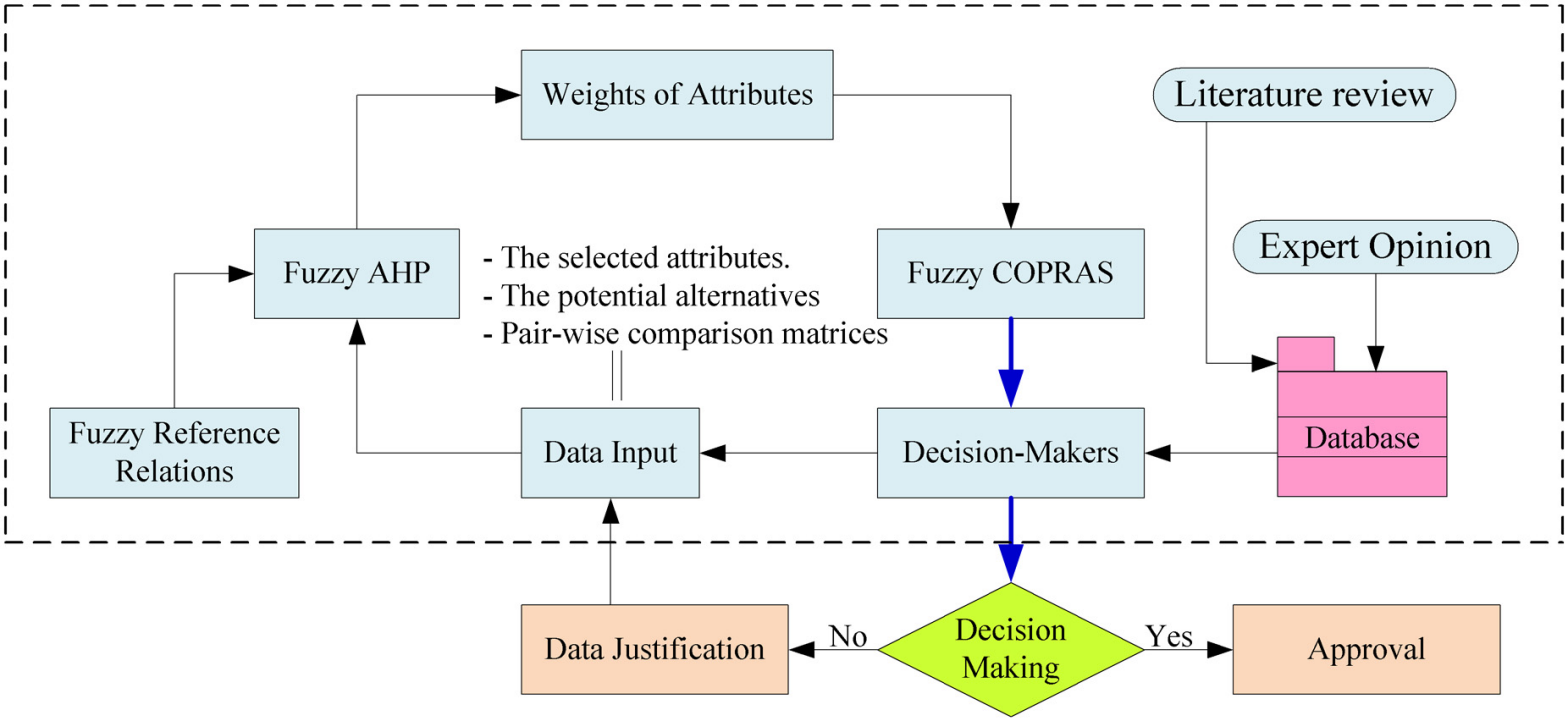
Scheme of the proposed model.

The structural hierarchy of the developed model is shown in Fig 1. The required data is initially prepared for the decision-making process. The database is collected from some sources such as literature, experts’ judgments and the catalogues of numerous manufacturers. Frequently meetings are organized to get feedback from the experts for the alternatives and attributes, and for the determination of data inputs for the fuzzy AHP with the preference relations. The priorities or weights of attributes are calculated by the improved fuzzy AHP with the pair-wise comparison matrix based on the experts’ judgments and fuzzy preference relations. The outputs of the improved fuzzy AHP are the inputs of the fuzzy COPRAS for determining the ranking of alternatives. The decision-makers can use this result for the decision-making process to choose the most suitable solution. If the result is not satisfactory, data justification should be carried out for inputs of improved fuzzy AHP and the final decision is determined by decision-makers.

**Fig 2:**
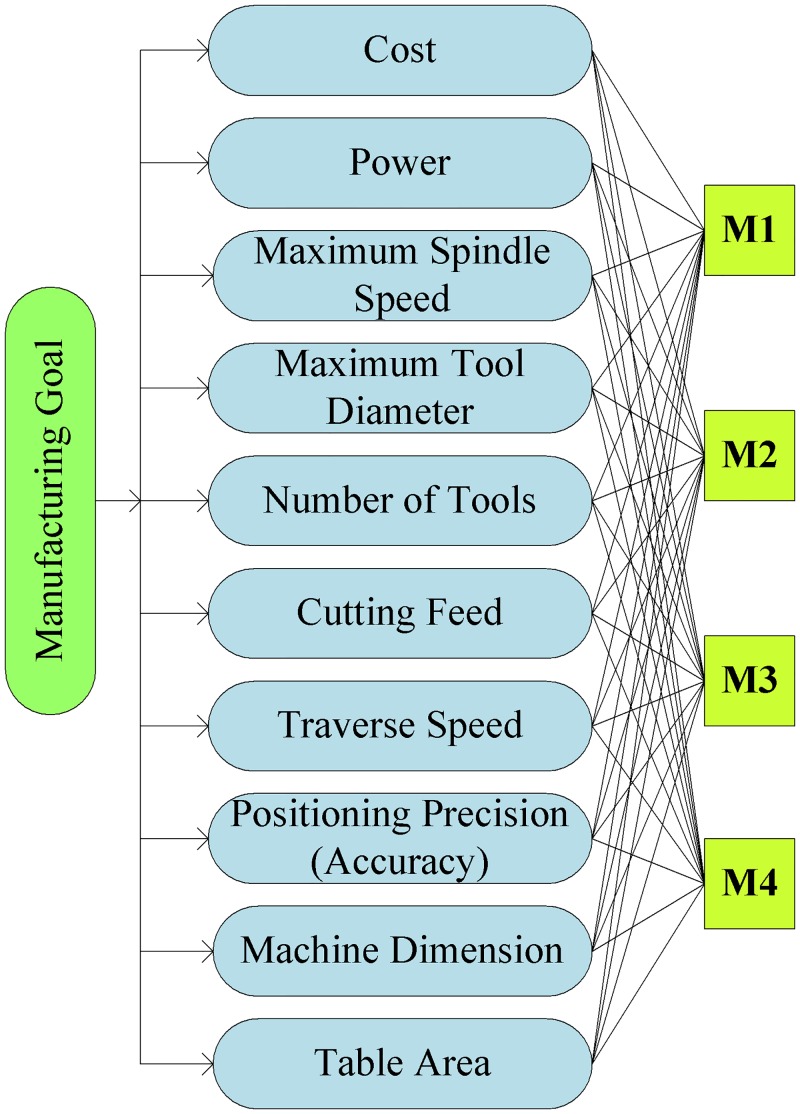
The hierarchical structure for machine tool evaluation.

The attributes in the decision support model are extracted from the literature, catalogues and interviews with experts in manufacturing. The hierarchical structure of the model is shown in Fig 2. It contains three top-down levels: At the first level (level 1), the manufacturing goal is determined for machine tool selection; the middle level (level 2) consists of attributes for the decision-making process such as Cost (A1), Power (A2), Maximum Spindle Speed (A3), Maximum Tool Diameter (A4), Number of Tools (A5), Cutting Feeds (A6), Traverse Speeds (A7), Positioning Precision/Accuracy (A8), Machine Dimensions (A9), and Table Area (A10).The candidate machine tools (M1, M2, M3, M4) are listed in the bottom level (level 3) for the ranking process.

### Methodology

**Fig 3:**
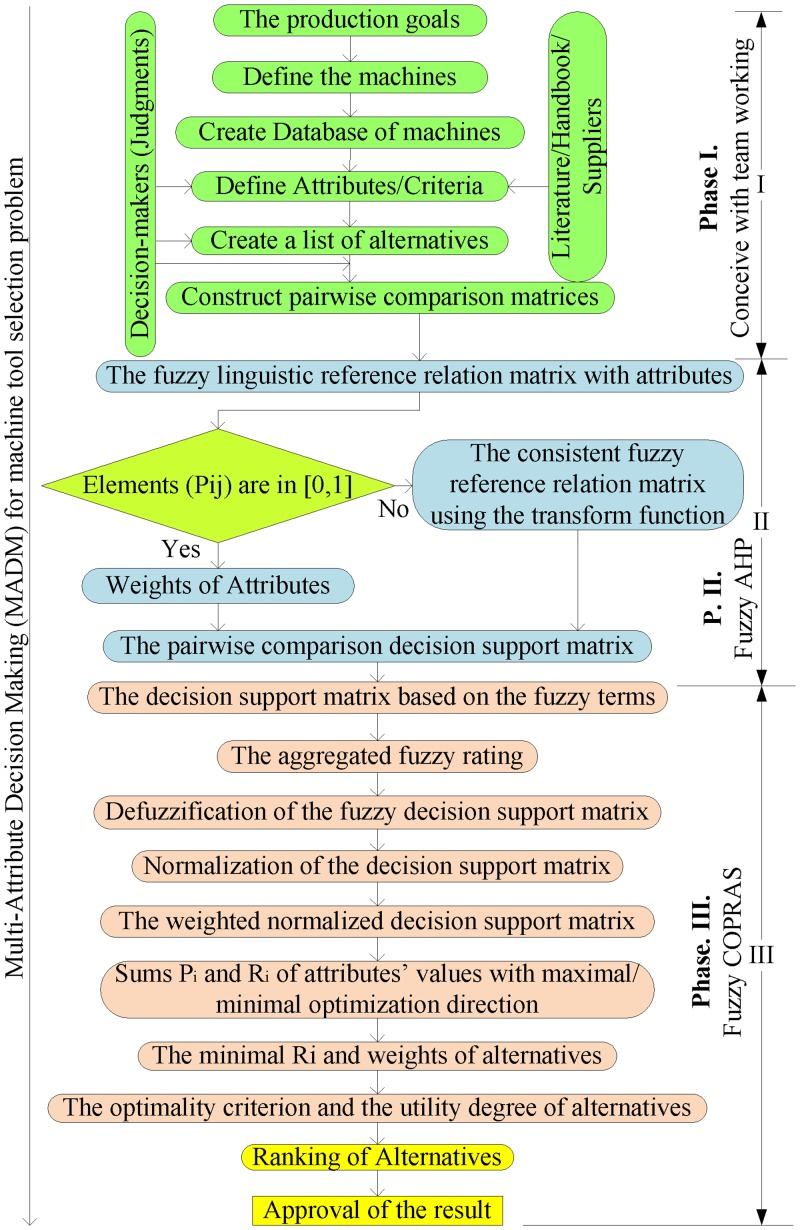
Flowchart of the proposed model.

The method developed for the decision-making process in machine tool evaluation is based on the combination of fuzzy AHP and fuzzy COPRAS. It makes use of the advantages of fuzzy AHP in determining the weights of attributes and the simplicity of fuzzy COPRAS for ranking alternatives. The integrated approach consists of three phases. In phase 1, a team work approach is taken to formulate the ideas for decision-making. In this stage, decision-makers define the attributes and alternatives from the market of machines or current manufacturing facilities. Information from handbook, past literatures and machine vendors are used to provide relevant knowledge and information for decision-makers to make accurate decisions in machine tool evaluation. The matrices of pair-wise comparisons are formulated from the attributes to prepare for the computation in phase 2 and phase 3. In phase 2, fuzzy AHP with linguistic preference relation is applied to determine the weights of attributes. Phase 3 inherits the results from phase 2 which are the weights of attributes in order to predict the weights of alternatives. The steps in phase 2 and phase 3 are shown in a flowchart of the proposed model (Fig 3).

### Fuzzy number

In complex evaluation systems, human’s judgments, knowledge and experiences are presented by linguistic terms and vague patterns. These are not presented as numbers but are defined as variables whose values are words or sentences in natural languages. These linguistic inputs can be represented quantitatively as a fuzzy number in various formats such as trapezoidal, triangular or Gaussian. In this study, the triangular fuzzy number (TFN) is used because of the intuitive and computational-efficient representation with straight lines in the membership function. Moreover, modeling using TFN was shown to be an effective approach for handling the decision problems involving vague and imprecise information [9, 14, 27].

Let $\tilde{A}$ be a fuzzy triangular number on ℜ,$\tilde{A}$ is defined as follows:$\tilde{A}=(l,m,u)$ if the membership function $\mu_{\tilde{A}}(x)$ satisfies the following rules:

$\mu_{\tilde{A}}(x):ℜ\to [0,1]$ and expressed as follows [51, 61]:

$$\mu_{\tilde{A}}(x)=\{\begin{array}{c} \frac{x-l}{m-l},l\leq x\leq m\\ \frac{u-x}{u-m},m\leq x\leq u\\ 0,otherwise \end{array}$$ (1)

**Fig 4:**
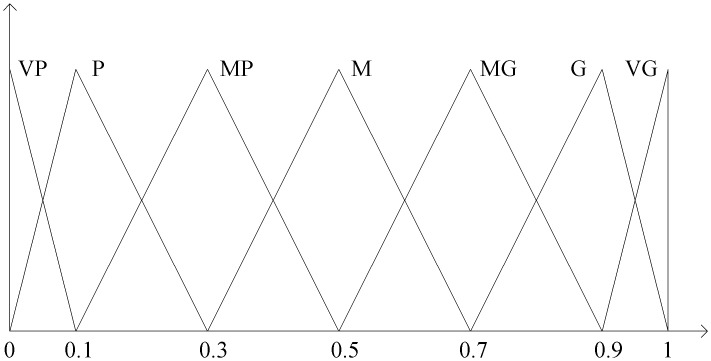
Fuzzy linguistic assessment variables [52].

We establish the linguistic description for the process of evaluation by interviewing the technicians, operators, managers and observations in the industry, utilizing a survey method in the engineering organizations. The linguistic variables are generated from expert’s experience and shown in Table 2 with seven levels of goodness. The membership functions of these linguistic variables are described in Fig 4. The use of linguistic variable is commonly utilized to measure the performance for each criterion based on expert’s judgments.

**Table 2:** Fuzzy linguistic assessment variables [5, 51].

Linguistic variables	Triangular fuzzy numbers (TFN)
Very poor (VP)	(0,0,0.1)
Poor (P)	(0,0.1,0.3)
Medium poor (MP)	(0.1,0.3,0.5)
Medium (M)	(0.3,0.5,0.7)
Medium good (MG)	(0.5,0.7,0.9)
Good (G)	(0.7,0.9,1)
Very Good (VG)	(0.9,1,1)

### Phase I: Conceive with team working

#### Step 1

Define the manufacturing goal for producing some desired types of part according to the customer’s demand.

#### Step 2

Define the machine tools which are necessary for formulating the manufacturing system in the manufacturing factory.

#### Step 3

Create a database of the machine tools from manufacturing supplier and the existing machine tools in the factory.

#### Step 4

Determine the desirable attributes implemented by decision-makers (DMs) for evaluating the machine tools.

#### Step 5

Choose the machine tool alternatives for the decision-making process.

#### Step 6

Build the hierarchical structure for decision-making process which presents the relationship of manufacturing goal, the attributes and alternatives in machine tool selection.

#### Step 7

Design of the questionnaire for data collection from experts’ judgments.

### Phase II: The AHP with consistent fuzzy reference relation [42, 52]

#### Step 8

Establish pair-wise comparison decision matrix $\tilde{A}$ based on the experts’ judgments for the attributes. Let A_i_ (i = 1, 2,…, n) be a set of attributes (a_ij_), and the relative importance between two attributes is evaluated using the TFNs:

$$\tilde{A}=\left[\begin{array}{cccc} 1 & {\tilde{a}}_{12} & \ldots & {\tilde{a}}_{1n}\\ {\tilde{a}}_{21} & 1 & \ldots & {\tilde{a}}_{2n}\\ \vdots & \vdots & \ddots & \vdots \\ {\tilde{a}}_{n1} & {\tilde{a}}_{n2} & \ldots & 1 \end{array}\right]=\left[\begin{array}{cccc} 1 & {\tilde{a}}_{12} & \ldots & {\tilde{a}}_{1n}\\ {\tilde{a}}_{12}^{-1} & 1 & \ldots & {\tilde{a}}_{2n}\\ \vdots & \vdots & \ddots & \vdots \\ {\tilde{a}}_{1n}^{-1} & {\tilde{a}}_{2n}^{-1} & \ldots & 1 \end{array}\right]$$ (2)

where ${\tilde{a}}_{ij}$ is a TFN or fuzzy linguistic variables, a_ij_ = (0.5, 0.5, 0.5) shows that no difference between i-th attribute and j-th attribute [52], which are presented in Table 2. The pair-wise comparison matrix ${\tilde{A}}_{n\times n}$ with n rows and n columns contains (nxn) elements, and the diagonal elements of matrix are known in advanced due to comparing with similar attributes. Thus, we have to determine (nxn-n) remaining elements in the matrix. Furthermore as the property of the pair-wise comparison is reciprocal, the values of the symmetry elements ${\tilde{a}}_{ij}^{-1}$ of matrix can be determined (in Appendix, Eq 7 and Eq 8). Thus, the number of judgments needed is n(n-1)/2. In addition, using the reciprocal fuzzy reference relation in Eqs 12–17 when value i and j run from 1 to (n-1) and (n-2), respectively, we can calculate the value of (n-1)(n-2)/2 elements in the matrix. The number of experts’ judgments required is n(n-1)/2-(n-1)(n-2)/2 = (n-1). Thus, the number of judgments in improved fuzzy AHP is significantly less than that of the normal fuzzy AHP [51, 52]. Other elements are determined based on the fuzzy preference relations as shown in Appendix (from Eqs 7–17).

#### Step 9

Construct the changed fuzzy pair-wise comparison decision matrix based on the fuzzy linguistic preference relations and transform functions. In this step, the transform function is employed to obtain the consistent fuzzy reference relation matrix from the decision matrix in Step 8. This means that after the pair-wise comparison decision matrix is determined, the value of some elements in the matrix are not in the interval [0,1] but would fall in an interval [-c, 1+c], (c>0 and c is the maximum amount of violation from the interval [0,1] among the elements of the decision matrix), the triangular fuzzy numbers obtained would be transformed using the transformation function (in Appendix, Eq 18) to preserve the reciprocity and addictive consistency [51, 52]. Table 3 shows the changed fuzzy pair-wise comparison matrix.

**Table 3:** The result of fuzzy linguistic reference relation matrix with the transforming function [51].

Goal	A_1_	A_2_	A_3_	…	A_n_	Average	Weights
A_1_	1	${\tilde{p}}_{12}$	${\tilde{p}}_{13}$	…	${\tilde{p}}_{1n}$	${\bar{A}}_{1}$	${\tilde{w}}_{a_{1}}$
A_2_	${\tilde{p}}_{12}^{-1}$	1	${\tilde{p}}_{23}$	…	${\tilde{p}}_{2n}$	${\bar{A}}_{2}$	${\tilde{w}}_{a_{2}}$
A_3_	${\tilde{p}}_{13}^{-1}$	${\tilde{p}}_{23}^{-1}$	1	…	${\tilde{p}}_{3n}$	${\bar{A}}_{3}$	${\tilde{w}}_{a_{3}}$
…	…	…	…	…	…	${\bar{A}}_{i}$	${\tilde{w}}_{a_{i}}$
A_n_	${\tilde{p}}_{1n}^{-1}$	${\tilde{p}}_{2n}^{-1}$	${\tilde{p}}_{3n}^{-1}$	…	1	${\bar{A}}_{n}$	${\tilde{w}}_{a_{n}}$

Where, ${\bar{A}}_{i}$ is the average of the values of the pair-wise comparison elements for each i-th row or each i-th attribute and ${\tilde{w}}_{a_{i}}$ is the weight of the i-th attribute.

$$\tilde{A}=\left[\begin{array}{cccc} 1 & {\tilde{p}}_{12} & \ldots & {\tilde{p}}_{1n}\\ {\tilde{p}}_{21} & 1 & \ldots & {\tilde{p}}_{2n}\\ \vdots & \vdots & \ddots & \vdots \\ {\tilde{p}}_{n1} & {\tilde{p}}_{n2} & \ldots & 1 \end{array}\right]=\left[\begin{array}{cccc} 1 & {\tilde{p}}_{12} & \ldots & {\tilde{p}}_{1n}\\ {\tilde{p}}_{12}^{-1} & 1 & \ldots & {\tilde{p}}_{2n}\\ \vdots & \vdots & \ddots & \vdots \\ {\tilde{p}}_{1n}^{-1} & {\tilde{p}}_{2n}^{-1} & \ldots & 1 \end{array}\right]$$ (3)

where ${\tilde{p}}_{ij}$ is the changed fuzzy number after using the transform function

$${\bar{A}}_{i}=\frac{1}{n}\sum_{j=1}^{n}p_{ij}=\left(\frac{1}{n}\sum_{j=1}^{n}p_{ij}^{L},\frac{1}{n}\sum_{j=1}^{n}p_{ij}^{M},\frac{1}{n}\sum_{j=1}^{n}p_{ij}^{R}\right)$$ (4)

$${\tilde{w}}_{a_{i}}=\left(w_{a_{i}}^{L},w_{a_{i}}^{M},w_{a_{i}}^{R}\right)=\frac{{\bar{A}}_{i}}{\sum_{i=1}^{m}{\bar{A}}_{i}}=\frac{\left(\frac{1}{n}\sum_{j=1}^{n}p_{ij}^{L},\frac{1}{n}\sum_{j=1}^{n}p_{ij}^{M},\frac{1}{n}\sum_{j=1}^{n}p_{ij}^{R}\right)}{{\bar{A}}_{1}+{\bar{A}}_{2}+\ldots +{\bar{A}}_{m}}$$ (5)

#### Step 10

Determine the defuzzied priorities/weights of the attributes using the simplest fuzzy mean [52].

$$w_{a_{i}}=\frac{w_{a_{i}}^{L}+w_{a_{i}}^{M}+w_{a_{i}}^{R}}{3}$$ (6)

### Phase III: Fuzzy COPRAS

In this phase, the procedure of fuzzy COPRAS described in Appendix A.2 is applied to determine the ranking of alternative. As a continuation from step 10, the steps 11–20 are adapted from the procedure of fuzzy COPRAS to calculate the weights of potential alternatives.

#### Step 11

**Fig 5:**
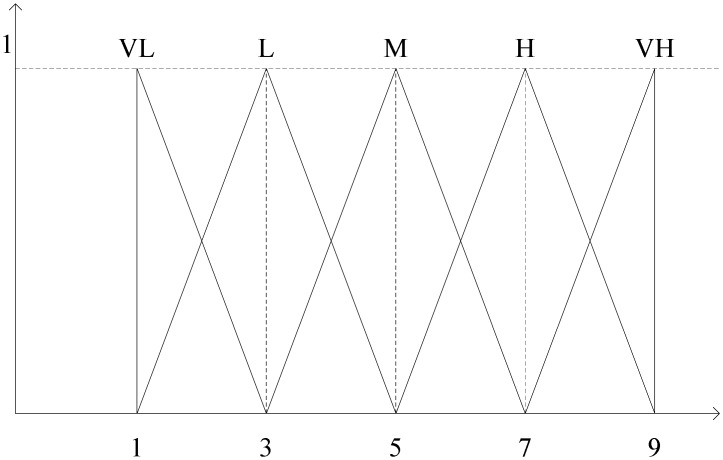
Linguistic variables for evaluating alternative.

Formulate the fuzzy decision support matrix/trade-off matrix using the fuzzy linguistic variables as reported in Table 4. The membership functions of fuzzy linguistic variables are described in Fig 5.

**Table 4:** Fuzzy linguistic variables.

Linguistic variable	TFN
Very Low (VL)	(1,1,3)
Low (L)	(1,3,5)
Medium (M)	(3,5,7)
High	(5,7,9)
Very High (VH)	(7,9,9)

#### Step 12

Defuzzification of the fuzzy trade-off matrix.

#### Step 13

Data normalization of the trade-off matrix.

#### Step 14

Determine the weighted normalized trade-off matrix.

#### Step 15

Calculate the total summation P_i_ (maximum optimization direction).

#### Step 16

Calculate the total summation R_i_ (minimum optimization direction).

#### Step 17

Determine the minimal of R_i_ value.

#### Step 18

Calculate the priority of each alternative.

#### Step 19

Determine the optimality criterion K.

#### Step 20

Calculate the utility degree of each alternative and determine the ranking.

## A Case Study for Machine Tool Selection

A case study is carried out for Keio Machining Lab with the assistance from experienced technicians having extensive knowledge in the field of machining processes. The selection of factors for decision-making is supervised by three experienced experts chosen based on the criteria as shown in Table 5. A survey for formulating the comparison decision matrix is conducted by the decision-makers with ten attributes (S1 File). These attributes are collected from past literatures and catalogues of CNC machines (cost-A1, power-A2, maximum spindle speed-A3, maximum tool diameter-A4, number of tools-A5, cutting feeds-A6, traverse speeds-A7, positioning precision accuracy-A8, machine dimensions-A9, table area-A10). They are described on the decision hierarchy, as in Fig 2. Four machines are chosen as alternatives from the potential suppliers for the decision-making. The matrix of pair-wise comparison between the attributes is filled with fuzzy linguistic assessment variables based on the expert’s judgments, as shown in Table 6.

**Table 5:** The characteristics of the three decision-making experts.

	Gender	Age	Education Level	Experience (years)	Job title	Job responsibility
**Decision-making expert 1 (DM1)**	Male	40–50	Bachelor in Manufacturing Engineering	>20	Manufacturing management and consultant at the supplier of CNC machine tools	Consultant in CNC machine tool and manufacturing process, production planning and scheduling.
**Decision-making expert 2 (DM2)**	Male	30–40	Bachelor in Mechanical Engineering	>10	Director of the manufacturing company	Management of manufacturing company, organization of production facilities and development of the machining process.
**Decision-making expert 3 (DM3)**	Male	40–50	Bachelor in Manufacturing Engineering	>20	Technician	Supervision of the machining process, determination of the machining parameters and control the CNC machines.

**Table 6:** Pair-wise comparison matrix among the attributes of CNC machines.

	A1	A2	A3	A4	A5	A6	A7	A8	A9	A10
**Cost (A1)**	*	M								
**Power (A2)**		*	P							
**Maximum Spindle Speed (A3)**			*	G						
**Maximum Tool Diameter (A4)**				*	MG					
**Number of Tools (A5)**					*	P				
**Cutting Feed (A6)**						*	G			
**Traverse Speed (A7)**							*	VP		
**Positioning Precision Accuracy (A8)**								*	VG	
**Machine Dimension (A9)**									*	P
**Table Area (A10)**										*

The (*) symbol in Table 5 presents the fuzzy number (0.5, 0.5, 0.5).

The comparison matrix among the attributes of machines is created with 9 elements/cells corresponding to 9 judgments from the expert. The rest of the elements within the matrix are calculated by applying Eqs 7–17 in the Appendix. A MATLAB program was developed to determine the values of the remainder of the elements in the decision matrix. The resulting elements are shown in Table 7. For example, to calculate the value of ${\tilde{p}}_{91}$ in the decision matrix, the equations Eqs 15–17 are utilized as follows.

**Table 7:** The fuzzy linguistic reference relation matrix with attributes.

	A1	A2	A3	A4	A5	A6	A7	A8	A9	A10
**A1**	(0.5,0.5,0.5)	(0.3,0.5,0.7)	(-0.2,0.1,0.5)	(0.0,0.5,1.0)	(0.0,0.7,1.4)	(-0.5,0.3,1.2)	(-0.3,0.7,1.7)	(-0.8,0.2,1.3)	(-0.4,0.7,1.8)	(-0.9,0.3,-0.5)
**A2**	(0.3,0.5,0.7)	(0.5,0.5,0.5)	(0.0,0.1,0.3)	(0.2,0.5,0.8)	(0.2,0.7,1.2)	(-0.3,0.3,1.0)	(-0.1,0.7,1.5)	(-0.6,0.2,1.1)	(-0.2,0.7,1.6)	(-0.7,0.3,1.4)
**A3**	(0.5,0.9,1.2)	(0.7,0.9,1.0)	(0.5,0.5,0.5)	(0.7,0.9,1.0)	(0.7,1.1,1.4)	(0.2,0.7,1.2)	(0.4,1.1,1.7)	(-0.1,0.6,1.3)	(0.3,1.1,1.8)	(-0.2,0.7,1.6)
**A4**	(0.0,0.5,1.0)	(0.2,0.5,0.8)	(0.0,0.1,0.3)	(0.5,0.5,0.5)	(0.5,0.7,0.9)	(0.0,0.3,0.7)	(0.2,0.7,1.2)	(-0.3,0.2,0.8)	(0.1,0.7,1.3)	(-0.4,0.3,1.1)
**A5**	(-0.4,0.3,1.0)	(-0.2,0.3,0.8)	(-0.4,-0.1,0.3)	(0.1,0.3,0.5)	(0.5,0.5,0.5)	(0.0,0.1,0.3)	(0.2,0.5,0.8)	(-0.3,0.0,0.4)	(0.1,0.5,0.9)	(-0.4,0.1,0.7)
**A6**	(-0.2,0.7,1.5)	(0.0,0.7,1.3)	(-0.2,0.3,0.8)	(0.3,0.7,1.0)	(0.7,0.9,1.0)	(0.5,0.5,0.5)	(0.7,0.9,1.0)	(0.2,0.4,0.6)	(0.6,0.9,1.1)	(0.1,0.5,0.9)
**A7**	(-0.7,0.3,1.3)	(-0.5,0.3,1.1)	(-0.7,-0.1,0.6)	(-0.2,0.3,0.8)	(0.2,0.5,0.8)	(0.0,0.1,0.3)	(0.5,0.5,0.5)	(0.0,0.0,0.1)	(0.4,0.5,0.6)	(0.4,0.6,0.9)
**A8**	(-0.3,0.8,1.8)	(-0.1,0.8,1.6)	(-0.3,0.4,1.1)	(0.2,0.8,1.3)	(0.6,1.0,1.3)	(0.4,0.6,0.8)	(0.9,1.0,1.0)	(0.5,0.5,0.5)	(0.9,1.0,1.0)	(0.4,0.6,0.8)
**A9**	(-0.8,0.3,1.4)	(-0.6,0.3,1.2)	(-0.8,-0.1,0.7)	(-0.3,0.3,0.9)	(0.1,0.5,0.9)	(-0.1,0.1,0.4)	(0.4,0.5,0.6)	(0.0,0.0,0.1)	(0.5,0.5,0.5)	(0.0,0.1,0.3)
**A10**	(1.5,0.7,1.9)	(-0.4,0.7,1.7)	(-0.6,0.3,1.2)	(-0.1,0.7,1.4)	(0.3,0.9,1.4)	(0.1,0.5,0.9)	(0.1,0.4,0.6)	(0.2,0.4,0.6)	(0.7,0.9,1.0)	(0.5,0.5,0.5)

$$\begin{array}{l} {\tilde{p}}_{91}=\left({\tilde{p}}_{91}^{L},{\tilde{p}}_{91}^{M},{\tilde{p}}_{91}^{R}\right)\\ p_{12}^{L}+p_{23}^{L}+p_{34}^{L}+p_{45}^{L}+p_{56}^{L}+p_{67}^{L}+p_{78}^{L}+p_{89}^{L}+p_{91}^{R}=\frac{(9-1)+1}{2}=\frac{9}{2}\\ \Rightarrow \{\begin{array}{c} p_{91}^{R}=\frac{9}{2}-(p_{12}^{L}+p_{23}^{L}+p_{34}^{L}+p_{45}^{L}+p_{56}^{L}+p_{67}^{L}+p_{78}^{L}+p_{89}^{L})\\ p_{91}^{M}=\frac{9}{2}-(p_{12}^{M}+p_{23}^{M}+p_{34}^{M}+p_{45}^{M}+p_{56}^{M}+p_{67}^{M}+p_{78}^{M}+p_{89}^{M})\\ p_{91}^{L}=\frac{9}{2}-(p_{12}^{R}+p_{23}^{R}+p_{34}^{R}+p_{45}^{R}+p_{56}^{R}+p_{67}^{R}+p_{78}^{R}+p_{89}^{R}) \end{array} \end{array}$$

Therefore, from the above equations, the value of element ${\tilde{p}}_{19}=\left(p_{19}^{L},p_{19}^{M},p_{19}^{R}\right)={\tilde{p}}_{91}^{-1}$. According to Eqs 9–11, we have:

$$p_{19}^{L}=1-p_{91}^{R};p_{19}^{M}=1-p_{91}^{M};p_{19}^{R}=1-p_{91}^{M}$$

Some elements of Table 7 fall outside the interval [0,1]. Thus, according to Eq 18, the transforming function f(x) = (x+0.9)/(1+2*0.9) is used to preserve the consistency of the matrix, and the result is shown in Table 8.

**Table 8:** Transforming results of the fuzzy linguistic reference relation matrix with function f(x) = (x+0.9)/(1+2x0.9).

	A1	A2	A3	A4	A5	A6	A7	A8	A9	A10
**A1**	(0.5,0.5,0.5)	(0.43,0.5,0.57)	(0.25,0.36,0.5)	(0.32,0.5,0.68)	(0.32,0.57,0.82)	(0.14,0.43,0.75)	(0.21,0.57,0.93)	(0.04,0.39,0.79)	(0.18,0.57,0.96)	(0.0,0.43,0.14)
**A2**	(0.43,0.5,0.57)	(0.5,0.5,0.5)	(0.32,0.36,0.43)	(0.39,0.5,0.61)	(0.39,0.57,0.75)	(0.21,0.43,0.68)	(0.29,0.57,0.86)	(0.11,0.39,0.71)	(0.25,0.57,0.89)	(0.07,0.43,0.82)
**A3**	(0.5,0.64,0.75)	(0.57,0.64,0.68)	(0.5,0.5,0.5)	(0.57,0.64,0.68)	(0.57,0.71,0.82)	(0.39,0.57,0.75)	(0.46,0.71,0.93)	(0.29,0.54,0.79)	(0.43,0.71,0.96)	(0.25,0.57,0.89)
**A4**	(0.32,0.5,0.68)	(0.39,0.5,0.61)	(0.32,0.36,0.43)	(0.5,0.5,0.5)	(0.5,0.57,0.64)	(0.32,0.43,0.57)	(0.39,0.57,0.75)	(0.21,0.39,0.61)	(0.36,0.57,0.79)	(0.18,0.43,0.71)
**A5**	(0.18,0.43,0.68)	(0.25,0.43,0.61)	(0.18,0.29,0.43)	(0.36,0.43,0.5)	(0.5,0.5,0.5)	(0.32,0.36,0.43)	(0.39,0.5,0.61)	(0.21,0.32,0.46)	(0.36,0.5,0.64)	(0.18,0.36,0.57)
**A6**	(0.25,0.57,0.86)	(0.32,0.57,0.79)	(0.25,0.43,0.61)	(0.43,0.57,0.68)	(0.57,0.64,0.68)	(0.5,0.5,0.5)	(0.57,0.64,0.68)	(0.39,0.46,0.54)	(0.54,0.64,0.71)	(0.36,0.5,0.64)
**A7**	(0.07,0.43,0.79)	(0.14,0.43,0.71)	(0.07,0.29,0.54)	(0.25,0.43,0.61)	(0.39,0.5,0.61)	(0.32,0.36,0.43)	(0.5,0.5,0.5)	(0.32,0.32,0.36)	(0.46,0.5,0.54)	(0.46,0.54,0.64)
**A8**	(0.21,0.61,0.96)	(0.29,0.61,0.89)	(0.21,0.46,0.71)	(0.39,0.61,0.79)	(0.54,0.68,0.79)	(0.46,0.54,0.61)	(0.64,0.68,0.68)	(0.5,0.5,0.5)	(0.64,0.68,0.68)	(0.46,0.54,0.61)
**A9**	(0.04,0.43,0.82)	(0.11,0.43,0.75)	(0.04,0.29,0.57)	(0.21,0.43,0.64)	(0.36,0.5,0.64)	(0.29,0.36,0.46)	(0.46,0.5,0.54)	(0.32,0.32,0.36)	(0.5,0.5,0.5)	(0.32,0.36,0.43)
**A10**	(0.86,0.57,1.0)	(0.18,0.57,0.93)	(0.11,0.43,0.75)	(0.29,0.57,0.82)	(0.43,0.64,0.82)	(0.36,0.5,0.64)	(0.36,0.46,0.54)	(0.39,0.46,0.54)	(0.57,0.64,0.68)	(0.5,0.5,0.5)

The average values and weights of attributes are determined with Eq 4 and Eq 5, and the defuzzification of fuzzy triangular numbers is calculated by Eq 6. Table 9 shows the results of average values, fuzzy weights and defuzzied weights of the attributes for the decision-making process.

**Table 9:** Weights of attributes.

	Average	Weights/Priorities	Defuzzied Weights
**A1**	(0.24,0.48,0.66)	(0.04,0.10,0.19)	0.1084
**A2**	(0.30,0.48,0.68)	(0.05,0.10,0.20)	0.1131
**A3**	(0.45,0.63,0.78)	(0.07,0.13,0.22)	0.1396
**A4**	(0.35,0.48,0.63)	(0.05,0.10,0.18)	0.1106
**A5**	(0.29,0.41,0.54)	(0.04,0.08,0.16)	0.0947
**A6**	(0.42,0.55,0.67)	(0.06,0.11,0.19)	0.1226
**A7**	(0.30,0.43,0.57)	(0.05,0.09,0.17)	0.0990
**A8**	(0.44,0.59,0.72)	(0.07,0.12,0.21)	0.1311
**A9**	(0.26,0.41,0.57)	(0.04,0.08,0.17)	0.0960
**A10**	(0.40,0.54,0.72)	(0.06,0.11,0.21)	0.1259
**Total**	(3.45,5.00,6.55)		

The decision matrix is established based on the experts’ judgments, as shown in Table 10. The experts use the fuzzy linguistic terms described in Table 4 to perform their assessment of each alternative against each attribute. Table 11 depicts the decision matrix with the presence of fuzzy numbers which has been converted from linguistic terms.

**Table 10:** Decision support matrix/trade-off matrix using fuzzy linguistic term in Table 4.

	A1	A2	A3	A4	A5	A6	A7	A8	A9	A10
**Machine 1 (MC1)**	H	L	H	M	M	M	M	VH	M	M
**Machine 2 (MC2)**	H	L	H	M	M	M	M	VH	M	M
**Machine 3 (MC3)**	H	L	M	H	VL	M	M	VH	M	M
**Machine 4 (MC4)**	H	L	M	H	VL	M	M	VH	M	M

**Table 11:** The trade-off matrix/decision matrix using the fuzzy numbers.

	A1	A2	A3	A4	A5	A6	A7	A8	A9	A10
**Machine 1 (MC1)**	(5,7,9)	(1,3,5)	(5,7,9)	(3,5,7)	(3,5,7)	(3,5,7)	(3,5,7)	(7,9,9)	(3,5,7)	(3,5,7)
**Machine 2 (MC2)**	(5,7,9)	(1,3,5)	(5,7,9)	(3,5,7)	(3,5,7)	(3,5,7)	(3,5,7)	(7,9,9)	(3,5,7)	(3,5,7)
**Machine 3 (MC3)**	(5,7,9)	(1,3,5)	(3,5,7)	(5,7,9)	(1,1,3)	(3,5,7)	(3,5,7)	(7,9,9)	(3,5,7)	(3,5,7)
**Machine 4 (MC4)**	(5,7,9)	(1,3,5)	(3,5,7)	(5,7,9)	(1,1,3)	(3,5,7)	(3,5,7)	(7,9,9)	(3,5,7)	(3,5,7)

In the subsequent step, defuzzification of the values of the elements or cells in the trade-off matrix is implemented using Eq 21. The results are shown in Table 12.

**Table 12:** Defuzzification of decision support matrix/trade-off matrix.

	A1	A2	A3	A4	A5	A6	A7	A8	A9	A10
**Machine 1 (MC1)**	7.00	3.00	7.00	5.00	5.00	5.00	5.00	8.33	5.00	5.00
**Machine 2 (MC2)**	7.00	3.00	7.00	5.00	5.00	5.00	5.00	8.33	5.00	5.00
**Machine 3 (MC3)**	7.00	3.00	5.00	7.00	1.67	5.00	5.00	8.33	5.00	5.00
**Machine 4 (MC4)**	7.00	3.00	5.00	7.00	1.67	5.00	5.00	8.33	5.00	5.00

After defuzzification of the trade-off matrix is implemented, the normalization values of the elements in the matrix are calculated according to step 6 in fuzzy COPRAS method. These are then converted to the weighted normalized values by multiplying by the weights of the attributes according to Eq 22. Finally, the weighted normalized decision support matrix is obtained as shown in Table 13.

**Table 13:** Weighted normalized decision matrix.

	A1	A2	A3	A4	A5	A6	A7	A8	A9	A10
**Weights**	0.1084	0.1131	0.1396	0.1106	0.0947	0.1226	0.0990	0.1311	0.0960	0.1259
**Optimization Direction**	Min	Max	Max	Max	Max	Max	Max	Max	Min	Max
**Machine 1 (MC1)**	0.1084	0.1131	0.1396	0.0790	0.0947	0.1226	0.0990	0.1311	0.0960	0.1259
**Machine 2 (MC2)**	0.1084	0.1131	0.1396	0.0790	0.0947	0.1226	0.0990	0.1311	0.0960	0.1259
**Machine 3 (MC3)**	0.1084	0.1131	0.0997	0.1106	0.0316	0.1226	0.0990	0.1311	0.0960	0.1259
**Machine 4 (MC4)**	0.1084	0.1131	0.0997	0.1106	0.0316	0.1226	0.0990	0.1311	0.0960	0.1259
**PIS**	0.1084	0.1131	0.1396	0.1106	0.0947	0.1226	0.0990	0.1311	0.0960	0.1259
**NIS**	0.1084	0.1131	0.0997	0.079	0.0316	0.1226	0.0990	0.1311	0.0960	0.1259
	A1: Cost					A6: Cutting Feed				
	A2: Power					A7: Traverse Speed				
	A3: Maximum Spindle Speed					A8: Position Precision				
	A4: Maximum Tool Diameter					A9: Machine Dimension				
	A5: Number of Tools					A10: Table Area				

In the following step, after the weighted normalized decision matrix is obtained, Eqs 23, 24, 25, 27 and 29 are used to determine the values of P_i_, R_i_, Q_i_, N_i_. The results are shown in Table 14. The PIS (Positive Ideal Solution) and NIS (Negative Ideal Solution) are used to determine the ranking according to TOPSIS methodology.

**Table 14:** The ranking for machine tool alternatives.

	Pi	Ri	Qi	Ni	Ranking	d(+)_Topsis_	d(-)_Topsis_	cc_Topsis_	Ranking_Topsis_
**Machine 1 (MC1)**	0.9050	0.2044	1.1094	100%	1	0.0100	0.0236	0.7026	1
**Machine 2 (MC2)**	0.9050	0.2044	1.1094	100%	2	0.0100	0.0236	0.7026	2
**Machine 3 (MC3)**	0.8336	0.2044	1.0380	93.565%	3	0.0236	0.0100	0.2974	3
**Machine 4 (MC4)**	0.8336	0.2044	1.0380	03.565%	3	0.0236	0.0100	0.2974	3

**Fig 6:**
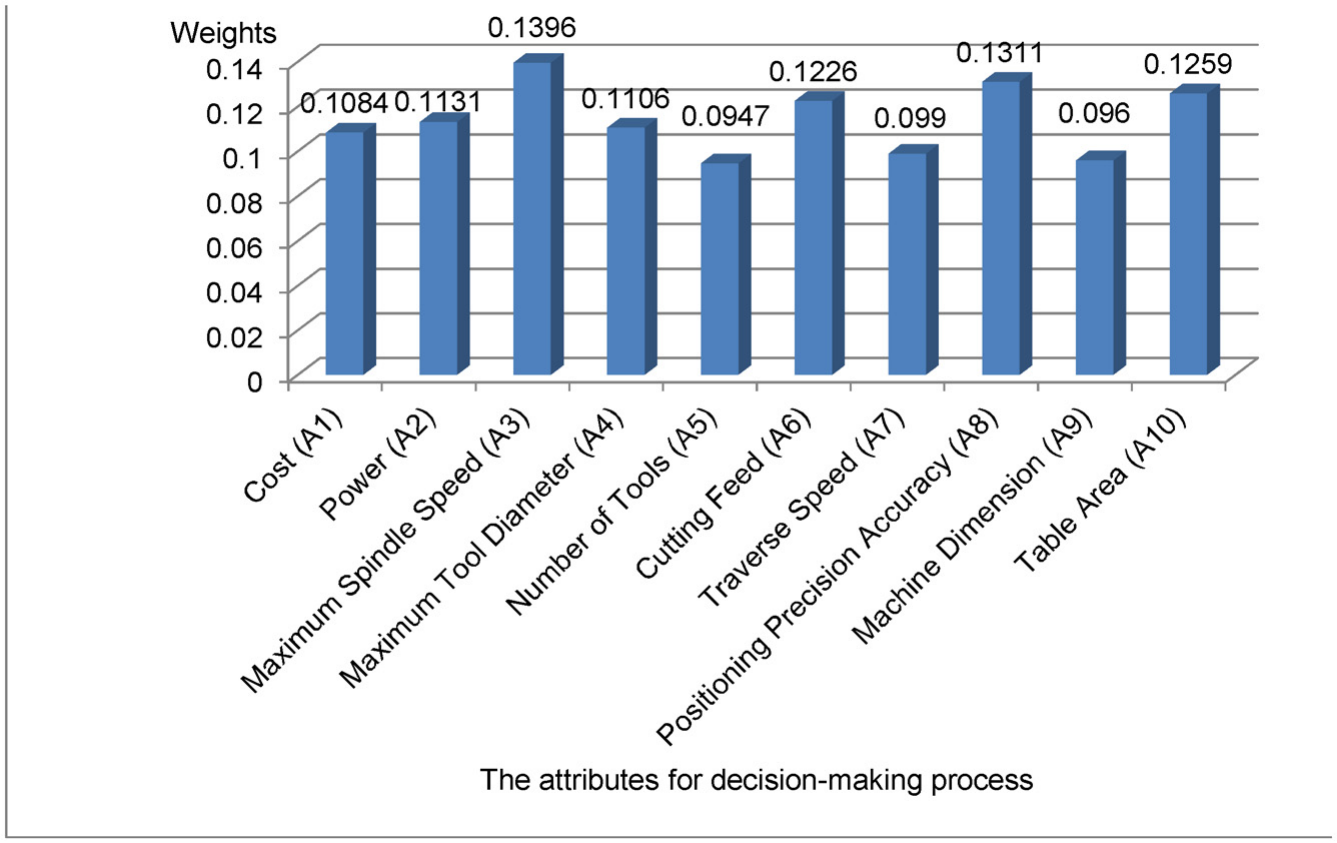
The weights/priorities of attributes.

**Fig 7:**
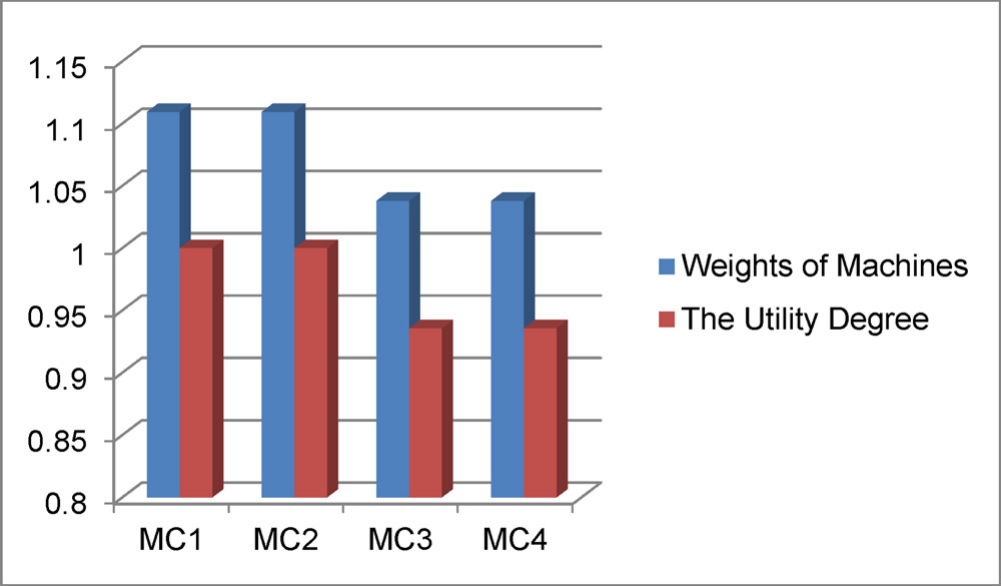
Ranking of alternatives.

The results from Table 14 and Figs 6 and 7 show that the ranking of alternatives is as follows: MC1 > MC2 > MC3 = MC4. Therefore, according to the collected data, we realize that MC1 is the best alternative with higher-ranking rate of the closeness coefficient for machine tool selection.

## Discussion

Evaluating machine tools evaluation for the implementation of manufacturing systems in production enterprises is a complex task which requires proper consideration in the technique and systems engineering management. The decision requires taking into account various factors to obtain the manufacturing goals and the capacity of the enterprise, and contains both a mixture of quantitative and qualitative factors. To overcome this problem, the model was developed based on the fuzzy AHP with consideration of fuzzy linguistic preference relation and fuzzy COPRAS to collect and analyze the judgments of experts for the selected attributes and the potential alternatives.

In this study, the MCDM model considered ten attributes for evaluating machine tools, as listed in Table 6. The weight of spindle speed is ranked the highest because this is a very important criterion to improve the productivity of manufacturing company. The second highest ranked criterion is the positioning precision accuracy to ensure the quality of product. Other significant criteria are table area, cutting feed, and power for improved productivity and the capacity for processing large-sized product. The cost of machine tool is also a concern for the small and medium enterprises. The final assignment of priority order for the attributes of machine tool is reasonable according to expert’s judgments, and it is also suitable for many cases in the practices at manufacturing companies. Four alternatives of CNC machine tools are selected and their ranking is determined based on fuzzy COPRAS based on weights from fuzzy AHP. This integrated approach has significantly reduced the required number of experts’ judgments.

The method of making decisions based on the experts’ judgments may the results in inconsistency, since it depends on the experience and knowledge of the decision-makers. Thus, results can differ when the different groups of experts are selected as evaluators. Thus, the aggregation of fuzzy sets is used to aggregate the experts’ judgments in the group. It is the duty of managers to carefully choose participants having the appropriate experience and knowledge. For example, in this study, the decision-maker have listed that the cutting feed is considered more important than the number of cutting tools. This shows that cutting performance may be appropriate for CNC machine considerations, but for a production system a greater number of cutting tools gives better system flexibility.

**Fig 8:**
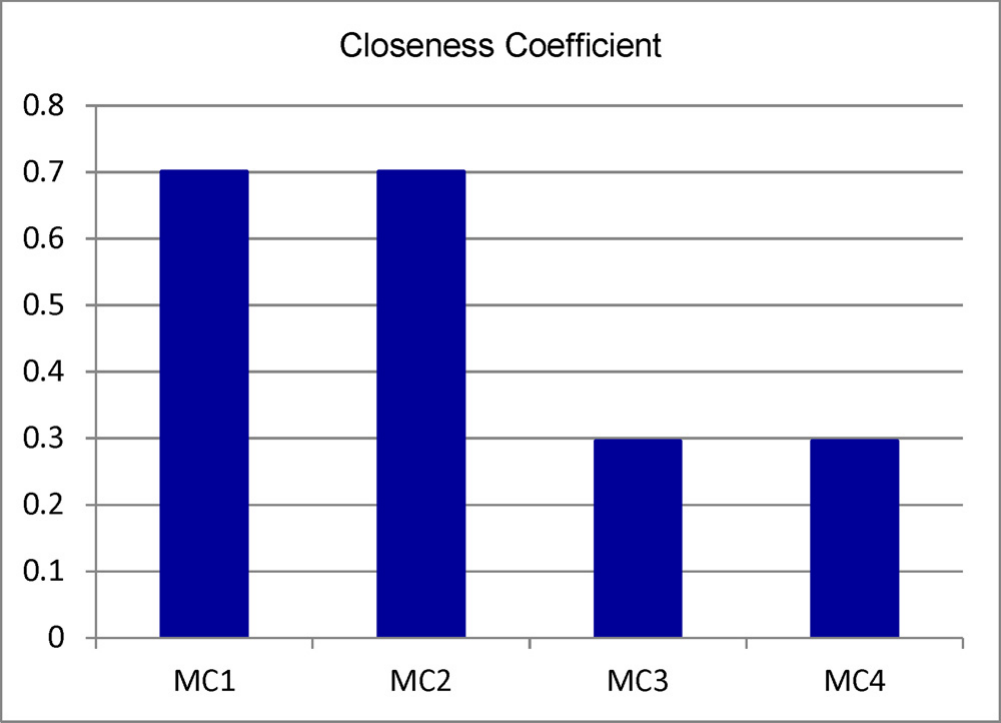
Closeness coefficient of machine tool alternatives.

The results of the proposed method shows that CNC machine 1 and CNC machine 3 have the same ranking. In this case the attributes need to be scrutinized more carefully, CNC machine 1 is better than CNC machine 2 as high-value attributes such maximum spindle speed (MC1: 10.000min^-1^ > MC2: 6000min^-1^). The result is validated with the classic TOPSIS method in Fig 8.

## Conclusion and Future Works

In today’s manufacturing environment, decision-making is a difficult and time-consuming process involving many attributes in today’s manufacturing environment. In most cases, these attributes can sometime imprecise and vague, and are difficult to be defined numerically. In this study, the integration of fuzzy AHP and Fuzzy COPRAS has shown significant advantage in data collection for processing uncertain information on machine tool evaluation. In particular, the fuzzy linguistic preference relation is used to determine the elements of decision matrix based on experts’ judgments. Using this approach, the number of expert judgment can be significant reduced while still ensuring the consistence of fuzzy AHP enabling a rapid decision-making process. This is a practical and applicable method for the decision-making process and helps engineers and managers to interpret information by modeling the quantitative and qualitative input data.

In this study, using the developed fuzzy AHP and fuzzy COPRAS, it was shown that MC1 is the best selection for implementation in manufacturing systems. However, the attributes of machine tools are hypothesized as independent factors affecting the decision-making without consideration of their interactions and inter-dependence. For future research work, the fuzzy ANP (Analytic Network Process) can be further developed and implemented based on fuzzy linguistic preference relations or its hybrid approaches with many different methods such fuzzy PROMETHEE, fuzzy ELECTRE, fuzzy VIKOR, fuzzy SAW, fuzzy ARAS, and fuzzy TOPSIS.

## Appendix

### A.1. Fuzzy reference relations

Definition 1 [51, 62, 63]: A fuzzy positive matrix

$$\tilde{A}=\left({\tilde{a}}_{ij}\right)\text{ is reciprocal }\Leftrightarrow {\tilde{a}}_{ji}={\tilde{a}}_{ij}^{-1}.$$ (7)

Definition 2 [51, 62]: A fuzzy positive matrix

A˜=(a˜ij) is consistent ⇔a˜ij⊗a˜jk≈a˜.ik (8)

Proposition 1 [42, 51, 52, 64]: Consider a set of alternatives, *X* = {*x*_1_,*x*_2_,…,*x*_*n*_} associated with a fuzzy reciprocal preference matrix $\tilde{A}=\left({\tilde{a}}_{ij}\right)$ with ${\tilde{a}}_{ij}\in \left[1/9,9\right]$ and the corresponding fuzzy reciprocal linguistic preference relation $\tilde{P}=\left({\tilde{p}}_{ij}\right)$ with ${\tilde{p}}_{ij}\in \left[0,1\right]$.

$$\text{a})\;p_{ij}^{L}+p_{ij}^{R}=1,\forall i,j\in \left\{1,2,\ldots ,n\right\}$$ (9)

$$\text{b})\;p_{ij}^{M}+p_{ij}^{M}=1,\forall i,j\in \left\{1,2,\ldots ,n\right\}$$ (10)

$$\text{c})\;p_{ij}^{R}+p_{ij}^{L}=1,\forall i,j\in \left\{1,2,\ldots ,n\right\}$$ (11)

Proposition 2 [42, 51, 52, 64]: For a reciprocal fuzzy reference relation $\tilde{P}=\left({\tilde{p}}_{ij}\right)=\left(p_{ij}^{L},p_{ij}^{M},p_{ij}^{R}\right)$ to be consistent, the following statement must be equivalent:

$$\text{a})\;p_{ij}^{L}+p_{jk}^{L}+p_{ki}^{R}=\frac{3}{2},\forall i<j<k$$ (12)

$$\text{b})\;p_{ij}^{M}+p_{jk}^{M}+p_{ki}^{M}=\frac{3}{2},\forall i<j<k$$ (13)

$$\text{c})\;p_{ij}^{R}+p_{jk}^{R}+p_{ki}^{L}=\frac{3}{2},\forall i<j<k$$ (14)

$$\text{d})\;p_{i(i+1)}^{L}+p_{\left(i+1)(i+2\right)}^{L}+\ldots +p_{(\text{j}-1)\text{j}}^{L}+p_{ji}^{R}=\frac{j-i+1}{2},\forall i<j$$ (15)

$$\text{e})\;p_{i(i+1)}^{M}+p_{\left(i+1)(i+2\right)}^{M}+\ldots +p_{(\text{j}-1)\text{j}}^{M}+p_{ji}^{M}=\frac{j-i+1}{2},\forall i<j$$ (16)

$$\text{f})\;p_{i(i+1)}^{R}+p_{\left(i+1)(i+2\right)}^{R}+\ldots +p_{(\text{j}-1)\text{j}}^{R}+p_{ji}^{L}=\frac{j-i+1}{2},\forall i<j$$ (17)

If the entries of the design matrix or the values of the matrix $\tilde{P}=\left({\tilde{p}}_{ij}\right)=\left(p_{ij}^{L},p_{ij}^{M},p_{ij}^{R}\right)$ are not in the interval [0, 1] but fall in an interval [-c, 1+c], (c>0), the obtained fuzzy numbers would need to be transformed by using transform function to preserve the reciprocity and addictive consistency; namely *f*:[-*c*,1+*c*]→[0,1].

$$f(x^{L,M,R})=\frac{x^{L,M,R}+c}{1+2c}$$ (18)

### A.2. The procedure of fuzzy COPRAS

The procedure of the fuzzy COPRAS includes the following steps:

Step 1. Define the linguistic terms used by decision-makers (Table 4).

Step 2. Construct the fuzzy decision support matrix. The preference ratios of alternatives are expressed by fuzzy linguistic variables in triangular fuzzy numbers.

Step 3. Determine the weights of the attributes.

Step 4. Calculate the aggregated fuzzy ratio ${\tilde{x}}_{i}{}_{j}$ of alternative *A*_*i*_ with respect to the attributes *C*_*j*_, where i = 1, 2, …, m and j = 1, 2, …, n.

$$D=\left[\begin{array}{ccccc} & C_{1} & C_{2} & \ldots & C_{n}\\ A_{1} & {\tilde{x}}_{11} & {\tilde{x}}_{12} & \ldots & {\tilde{x}}_{1n}\\ A_{2} & {\tilde{x}}_{21} & {\tilde{x}}_{22} & \ldots & {\tilde{x}}_{2n}\\ \vdots & \vdots & \vdots & \ddots & \vdots \\ A_{m} & {\tilde{x}}_{m1} & {\tilde{x}}_{m2} & \ldots & {\tilde{x}}_{mn} \end{array}\right],\;$$ (19)

where i = 1, 2, …, m; and j = 1, 2, …, n.

$${\tilde{x}}_{ij}=(x_{ij1},x_{ij2},x_{ij3}),$$

$$x_{ij1}=\text{min}\left\{x_{ijk1}\right\};x_{ij2}=\frac{1}{K}\sum_{k=1}^{K}x_{ijk2};x_{ij3}=\text{max}\left\{x_{ijk3}\right\}$$ (20)

where ${\tilde{x}}_{ijk}$ is the ratio of alternative *A*_*i*_ with respect to the attribute *C*_*j*_ evaluated by k-th expert (K is a number of experts)${\tilde{x}}_{ijk}=(x_{ijk1},x_{ijk2},x_{ijk3})$.

Step 5. Defuzzification of the aggregated fuzzy decision support matrix:

After aggregating the fuzzy scale in the fuzzy decision support matrix is completed, the matrix D is converted into the aggregated fuzzy decision support matrix and then the defuzzification of this matrix is implemented to obtain the crisp values by applying the center of area method by the following equation [59, 65, 66]. In particular, U, M and L are upper, medium and lower limitation of fuzzy number x, respectively.

$$x_{ij}=\frac{\left[\left(Ux_{ij}-Lx_{ij})+(Mx_{ij}-Lx_{ij}\right)\right]}{3}+Lx_{ij}$$ (21)

Step 6. Normalize the data in the decision support matrix (*f*_*ij*_). The normalization of the decision-making process is implemented by determining the ratio of the value of each attribute and the largest value in each column to transform the values of the attributes into value boundary [0, 1] and all the attributes are dimensionless.

Step 7. Determine the weighted normalized decision support matrix $\left({\hat{x}}_{ij}\right)$ through each element/cell in the matrix. It is calculated by multiplying the priority weight of the selected attribute (*w*_*j*_) with the respective normalized value in the decision support matrix:

$${\hat{x}}_{ij}=f_{ij}\times w_{j}$$ (22)

Step 8. Calculate the total summation P_i_ of the values of the attributes with the desire of achieving the greatest value in the maximal optimization direction for each alternative (line/row of the decision support matrix):

$$P_{j}=\sum_{j=1}^{k}{\hat{x}}_{ij}$$ (23)

Step 9. Calculate the total summation R_i_ of the values of the attributes with the desire of achieving the smallest value in the minimal optimization direction for each alternative (line/row of the decision support matrix):

$$R_{i}=\sum_{j=k+1}^{m}{\hat{x}}_{ij}$$ (24)

In the above formula, there (m-k) attributes need to be minimized.

Step 10. Determine the minimal value of R_i_:

$$R_{\text{min}}=\text{min}\{R_{i}\},\;i\;=\;1,\;2,\;\ldots ,\;n.$$ (25)

Step 11. Determine the priority weight of each alternative *Q*_*i*_:

$$Q_{i}=P_{i}+\frac{R_{\text{min}}\sum_{i=1}^{n}R_{i}}{R_{i}\sum_{i=1}^{n}\frac{R_{\text{min}}}{R_{i}}}$$ (26)

The above formula can be written as follows:

$$Q_{i}=P_{i}+\frac{\sum_{i=1}^{n}R_{i}}{R_{i}\sum_{i=1}^{n}\frac{1}{R_{i}}}$$ (27)

Step 12. Calculate the optimality criterion K:

$$K=\text{max}\{Q_{i}\},\;i\;=\;1,\;2,\;\ldots ,\;n.$$ (28)

Step 13. Assignment of the priority of the alternatives. The greater the priority weight of alternative *Q*_*i*_, the higher is the rank of the alternative. Therefore, the alternative with Q_max_ value is the most suitable selection in the decision-making process, which obtains the highest satisfaction degree.

Step 14. Determine the utility degree of each alternative:

$$N_{i}=\frac{Q_{i}}{Q_{\text{max}}}\times 100\%$$ (29)

where *Q*_*i*_ and *Q*_*max*_ are the weight of alternatives obtained from the above equation.

## Supporting Information

S1 File Questionnaire design for decision-making in machine tool evaluation. (PDF)

## References

[1] Taha Z, Rostam S. A fuzzy AHP–ANN-based decision support system for machine tool selection in a flexible manufacturing cell. The International Journal of Advanced Manufacturing Technology. 2011; 57(5–8): 719–733.

[2] Turskis Z, Zavadskas EK, Kutut V. A Model Based on ARAS-G and AHP Methods for Multiple Criteria Prioritizing of Heritage Value. International Journal of Information Technology & Decision Making. 2013; 12: 45–73.

[3] Okul D, Gencer C, Aydogan EK. A Method Based on SMAA-Topsis for Stochastic Multi-Criteria Decision Making and a Real-World Application. International Journal of Information Technology & Decision Making. 2014; 13: 957–978.

[4] Arslan M, Catay B, Budak E. A decision support system for machine tool selection. Journal of Manufacturing Technology Management. 2004; 15(1): 101–109.

[5] Ertuğrul İ, Güneş M. Fuzzy Multi-criteria Decision Making Method for Machine Selection. Analysis and Design of Intelligent Systems using Soft Computing Techniques, eds. Melin Patricia, Castillo Oscar, Ramirez EduardoGomez, Kacprzyk Janusz and Pedrycz Witold, Advances in Soft Computing (Springer Berlin Heidelberg, 2007), pp. 638–648.

[6] Chung C-H. Loading flexible manufacturing systems: a heuristic approach. Computers & Industrial Engineering. 1986; 11(1–4): 246–250.

[7] Stecke KE. Formulation and solution of nonlinear integer production planning problems for flexible manufacturing systems. Management Science. 1983; 29(3): 273–288.

[8] Abdel-Malek L, Resare LJ. Algorithm based decision support system for the concerted selection of equipment in machining/assembly cells. International Journal of Production Research. 2000; 38(2): 323–339.

[9] Ayağ Z, Özdemir R. A fuzzy AHP approach to evaluating machine tool alternatives. Journal of Intelligent Manufacturing. 2006; 17(2): 179–190.

[10] Önüt S, Soner Kara S, Efendigil T. A hybrid fuzzy MCDM approach to machine tool selection. Journal of Intelligent Manufacturing. 2008; 19(4): 443–453.

[11] Ayağ Z. A hybrid approach to machine-tool selection through AHP and simulation. International Journal of Production Research. 2007; 45(9): 2029–2050.

[12] Taha Z, Rostam S. A hybrid fuzzy AHP-PROMETHEE decision support system for machine tool selection in flexible manufacturing cell. Journal of Intelligent Manufacturing. 2011; 23(6): 2137–2149.

[13] Dağdeviren M. Decision making in equipment selection: an integrated approach with AHP and PROMETHEE. Journal of Intelligent Manufacturing. 2008; 19(4): 397–406.

[14] Durán O, Aguilo J. Computer-aided machine-tool selection based on a Fuzzy-AHP approach. Expert Systems with Applications. 2008; 34(3): 1787–1794.

[15] Abdi M. Fuzzy multi-criteria decision model for evaluating reconfigurable machines. International Journal of Production Economics. 2009; 117(1): 1–15.

[16] Ic YT, Yurdakul M, Eraslan E. Development of a component-based machining centre selection model using AHP. International Journal of Production Research. 2012; 50(22): 6489–6498.

[17] Lin ZC, Yang CB. Evaluation of machine selection by the AHP method. Journal of Materials Processing Technology. 1996; 57(3–4): 253–258.

[18] İç YT, Yurdakul M. Development of a decision support system for machining center selection. Expert Systems with Applications. 2009; 36(2): 3505–3513.

[19] Qi J. Machine tool selection model based on fuzzy MCDM approach Proceedings of 2010 International Conference on Intelligent Control and Information Processing (ICICIP), IEEE, 2010, pp. 282–285.

[20] Hasan Aghdaie M, Hashemkhani Zolfani S, Zavadskas EK. Decision making in machine tool selection: An integrated approach with SWARA and COPRAS-G methods. Engineering Economics. 2013; 24(1): 5–17.

[21] Krylovas A, Zavadskas EK, Kosareva N, Dadelo S. New KEMIRA Method for Determining Criteria Priority and Weights in Solving MCDM Problem. International Journal of Information Technology & Decision Making. 0: 1–15, 10.1142/S0219622014500825

[22] Myint S, Tabucanon MT. A multiple-criteria approach to machine selection for flexible manufacturing systems. International Journal of Production Economics. 1994; 33(1–3): 121–131.

[23] Tabucanon MT, Batanov DN, Verma DK. Decision support system for multicriteria machine selection for flexible manufacturing systems. Computers in industry. 1994; 25(2): 131–143.

[24] Yurdakul M. AHP as a strategic decision-making tool to justify machine tool selection. Journal of Materials Processing Technology. 2004; 146(3): 365–376.

[25] Samvedi A, Jain V, Chan FTS. An integrated approach for machine tool selection using fuzzy analytical hierarchy process and grey relational analysis. International Journal of Production Research. 2011; 50(12): 3211–3221.

[26] Paramasivam V, Senthil V, Rajam Ramasamy N. Decision making in equipment selection: an integrated approach with digraph and matrix approach, AHP and ANP. The International Journal of Advanced Manufacturing Technology. 2011; 54(9): 1233–1244.

[27] Ayağ Z, Gürcan Özdemir R. Evaluating machine tool alternatives through modified TOPSIS and alpha-cut based fuzzy ANP. International Journal of Production Economics. 2012; 140(2): 630–636.

[28] Ayağ Z, Özdemir R, An intelligent approach to machine tool selection through fuzzy analytic network process. Journal of Intelligent Manufacturing. 2011; 22(2): 163–177.

[29] Nguyen HT, Dawal SZM, Nukman Y, Aoyama H. A hybrid approach for fuzzy multi-attribute decision making in machine tool selection with consideration of the interactions of attributes. Expert Systems with Applications. 2014; 41(6): 3078–3090.

[30] Chakraborty S. Applications of the MOORA method for decision making in manufacturing environment. The International Journal of Advanced Manufacturing Technology. 2011; 54(9): 1155–1166.

[31] Ozgen A, Tuzkaya G, Tuzkaya UR, Ozgen D. A Multi-Criteria Decision Making Approach for Machine Tool Selection in a Fuzzy Environment. International Journal of Computational Intelligence Systems. 2011; 4(4): 431–445.

[32] Tsai JP, Cheng HY, Wang SY, Kao YC, Multi-criteria decision making method for selection of machine tool Proceedings of 2010 International Symposium on Computer Communication Control and Automation (3CA), IEEE, 2010, pp. 49–52.

[33] Yurdakul M, İç YT. Analysis of the benefit generated by using fuzzy numbers in a TOPSIS model developed for machine tool selection problems. Journal of Materials Processing Technology. 2009; 209(1): 310–317.

[34] Balaji CM, Gurumurthy A, Kodali R. Selection of a machine tool for FMS using ELECTRE III—a case study, Proceedings of the IEEE International Conference on Automation Science and Engineering CASE, 2009, pp. 171–176.

[35] Sun B, Chen H, Du L, Fang Y. Machine Tools Selection Technology for Networked Manufacturing, Proceedings of the IEEE International Symposium on Intelligent Information Technology and Application, IITA '08, 2008, pp. 530–534.

[36] Rao RV. Machine group selection in a flexible manufacturing cell using digraph and matrix methods. International Journal of Industrial and Systems Engineering. 2006; 1(4): 502–518.

[37] Rao RV. Decision Making in the Manufacturing Environment using Graph Theory and Fuzzy Multiple Attribute Decision Making, Chapter 10: Machine Selection in a Flexible Manufacturing Cell, Advanced Manufacturing (Springer-Verlag London Limited, 2007), pp. 139–148.

[38] Chtourou H, Masmoudi W, Maalej A. An expert system for manufacturing systems machine selection. Expert Systems with Applications. 2005; 28(3): 461–467.

[39] Wang TY, Shaw CF, Chen YL. Machine selection in flexible manufacturing cell: a fuzzy multiple attribute decision-making approach. International Journal of Production Research. 2000; 38(9): 2079–2097.

[40] Peng Y, Kou G, Wang G, Wu W, Shi Y. Ensemble of software defect predictors: an AHP-based evaluation method. International Journal of Information Technology & Decision Making. 2011; 10: 187–206.

[41] Yi X & Wang L. Land Suitability Assessment on a Watershed of Loess Plateau Using the Analytic Hierarchy Process. PloS one. 2013; 8(7), e69498 10.1371/journal.pone.0069498 23922723 PMC3724925

[42] Chen Y-H, Chao R-J. Supplier selection using consistent fuzzy preference relations. Expert Systems with Applications. 2012; 39(3): 3233–3240.

[43] Wu L-T, Cui X, Dai R-W. Judgment number reduction: An issue in the analytic hierarchy process. International Journal of Information Technology & Decision Making. 2010; 9: 175–189.

[44] Nieto-Morote A, Ruz-Vila F. A Fuzzy AHP Multi-Criteria Decision-Making Approach Applied to Combined Cooling, Heating, and Power Production Systems. International Journal of Information Technology & Decision Making. 2011; 10: 497–517.

[45] Xu Y, Patnayakuni R, Wang H. A Method based on Mean Deviation for Weight Determination from Fuzzy Preference Relations and Multiplicative Preference Relations. International Journal of Information Technology & Decision Making. 2012; 11: 627–641.

[46] Franco CA. On the analytic hierarchy process and decision support based on fuzzy-linguistic preference structures. Knowledge-Based Systems. 2014; 70: 203–211.

[47] Meng F, Chen X. A new method for group decision making with incomplete fuzzy preference relations. Knowledge-Based Systems. 2014; 10.1016/j.knosys.2014.09.011

[48] Yan HB, Ma T. A group decision-making approach to uncertain quality function deployment based on fuzzy preference relation and fuzzy majority. European Journal of Operational Research. 2014; 10.1016/j.ejor.2014.09.017

[49] Tao Z, Liu X, Chen H & Chen Z. Group decision making with fuzzy linguistic preference relations via cooperative games method. Computers & Industrial Engineering. 2015; 83(0), 184–192. 10.1016/j.cie.2015.02.016.

[50] Deng X, Lu X, Chan FTS, Sadiq R, Mahadevan S & Deng Y. D-CFPR: D numbers extended consistent fuzzy preference relations. Knowledge-Based Systems. 2015; 73(0), 61–68. 10.1016/j.knosys.2014.09.007

[51] Wang T-C, Chen Y-H. Applying fuzzy linguistic preference relations to the improvement of consistency of fuzzy AHP. Information Sciences. 2008; 178(19): 3755–3765.

[52] Rezaei J, Ortt R. Multi-criteria supplier segmentation using a fuzzy preference relations based AHP. European Journal of Operational Research. 2013; 225(1): 75–84.

[53] Hajiagha SHR, Mahdiraji HA, Zavadskas EK, Hashemi SS. Fuzzy Multi-Objective Linear Programming Based on Compromise VIKOR Method. International Journal of Information Technology & Decision Making. 2014; 13(4): 679–698.

[54] Liao C-N. A Fuzzy Approach to Business Travel Airline Selection using an Integrated AHP-TOPSIS-MSGP Methodology. International Journal of Information Technology & Decision Making. 2013; 12(1): 119–137.

[55] Chang K-H, Chang Y-C, Lee Y-T, Integrating TOPSIS and DEMATEL Methods to Rank the Risk of Failure of FMEA. International Journal of Information Technology & Decision Making. 2014; 0: 1–29, 10.1142/S0219622014500758

[56] Zavadskas EK, Vainiunas P, Turskis Z, Tamosaitiene J. Multiple criteria decision support system for assessment of projects managers in construction. International Journal of Information Technology & Decision Making. 2012; 11(2): 501–520.

[57] Zavadskas E, Kaklauskas A. Determination of an efficient contractor by using the new method of multicriteria assessment, International Symposium for “The Organization and Management of Construction”. Shaping Theory and Practice, 1996, pp. 94–104.

[58] Chatterjee N, Bose G. A COPRAS-F base multi-criteria group decision making approach for site selection of wind farm. Decision Science Letters. 2013; 2(1): 1–10.

[59] Fouladgar MM, Yazdani-Chamzini A, Zavadskas EK, Haji Moini SH, A new hybrid model for evaluating the working strategies: case study of construction company. Technological and Economic Development of Economy. 2012; 18(1): 164–188.

[60] Yazdani M, Alidoosti A, Kazimieras Zavadskas E. Risk analysis of critical infrastructures using fuzzy COPRAS. Economic Research. 2011; 24(4): 27–40.

[61] Klir G, Yuan B. Fuzzy sets and fuzzy logic (Prentice Hall New Jersey, 1995).

[62] Buckley JJ. Fuzzy hierarchical analysis. Fuzzy Sets and Systems. 1985; 17(3):233–247.

[63] Ekel PY, Silva MR, Schuffner Neto F, Palhares RM. Fuzzy preference modeling and its application to multiobjective decision making. Computers & Mathematics with Applications. 2006; 52(1–2): 179–196.

[64] Chen Y-H, Wang T-C, Wu C-Y. Multi-criteria decision making with fuzzy linguistic preference relations. Applied Mathematical Modelling. 2011; 35(3): 1322–1330.

[65] Wu H-Y, Tzeng G-H, Chen Y-H. A fuzzy MCDM approach for evaluating banking performance based on Balanced Scorecard. Expert Systems with Applications. 2009; 36(6): 10135–10147.

[66] Chang T-H, Wang T-C. Using the fuzzy multi-criteria decision making approach for measuring the possibility of successful knowledge management. Information Sciences. 2009; 179(4): 355–370.

