# Supplementary material for: An Integrated Approach of Fuzzy Linguistic Preference Based AHP and Fuzzy COPRAS for Machine Tool Evaluation
Source: PLoS One. 2015 Sep 14;10(9):e0133599. doi: 10.1371/journal.pone.0133599 (PMC4569346; doi:10.1371/journal.pone.0133599)
Supplement: S1 File — (PDF) [file pone.0133599.s001.pdf]

Specification of CNC machine

Machine model	MC ①	MC ②	NCF ①	NCF ②	
Attributes/Machines	Machine 1	Machine 2	Machine 3	Machine 4	Machine 5
Cost	1,200 万円	1,200 万円	1,000 万円		
Power	3.7 kW 連続定格<br>5.5 kW 30分定格	〃	3.7kW (連続)<br>5.5kW (30分)		
Maximum spindle speed	10000 min^{-1}	6000 min^{-1}	4000 min^{-1}		
Maximum tool diameter	Φ76.2 × 300 mm	〃	?		
Number of tools	30	20	—	〃	
Cutting feed	10000 mm/min	〃	X,Y 4000 mm/min<br>Z 2000 mm/min		
Traverse speed	15000 mm/min	〃	X,Y 4000 mm/min<br>Z 2000 mm/min		
Positioning Precision (accuracy)	0.001 mm	〃	0.001 mm		
Machine dimension	W D H<br>2515×2635×2450	〃	W D H<br>1880×2080×2090		
Table area	800 × 350	〃	800 × 320		

The purpose of questionnaire design is to determine the weight priorities of the selected attributes for multi-attribute decision-making process in the most suitable CNC machine tool selection to implement a flexible manufacturing cell, satisfies the Small and Medium Enterprise's (SME) manufacturing goals is to produce few types of parts. The results is only used for the academic research purpose. The questionnaire should be completed by the experienced experts understanding the CNC machine tools as well as manufacturing system and technology.

The following questions refer a questionnaire hierarchical structure (table below) to determine the importance of the attributes and the weight priorities of alternatives by **putting check marks** on the pair-wise comparison matrices. An example is shown below.

Question: **How important is "cost " attribute when it is compared with "power" attribute for machine tool selection**.

1=equal important (0.3,0.5,0.7)

3=Moderately important (0.5,0.7,0.9)

5=Strongly important (0.7,0.9,1)

7=Very strongly important (0.9,1,1)

Questionnaire form used to compare the machine tool selection attributes.

If we want to choose a CNC machine tool to buy for investing the production systems in companies. Which the attributes are the most important in your choice for decision-making in machine selection. Please fill in the form for your opinion/judgments.

Cost	7	5	3	(1)	3	5	7	Power
Power	7	5	3	1	3	(5)	7	Maximum spindle speed
Maximum spindle speed	7	(5)	3	1	3	5	7	Maximum tool diameter
Maximum tool diameter	7	5	(3)	1	3	5	7	Number of tools
Number of tools	7	5	3	1	3	(5)	7	Cutting feed
Cutting feed	7	(5)	3	1	3	5	7	Traverse speed
Traverse speed	7	5	3	1	3	5	(7)	Positioning Precision (accuracy)
Positioning Precision (accuracy)	(7)	5	3	1	3	5	7	Machine dimension
Machine dimension	7	5	3	1	3	(5)	7	Table area
Table area	7	5	3	(1)	3	5	7	cost

Section 2: **Evaluating the attributes for each machine**

Linguistic variables (attribute/alternative)	Triangular fuzzy scale
Very Low (VL)	(1,1,3)
Low (L)	(1,3,5)
Medium (M)	(3,5,7)
High (H)	(5,7,9)
Very High (VH)	(7,9,9)

According to your opinion, How attribute for each machine. Please mark (VL, L, M, H or VH) the below table.
For example: Machine 1 has a very high cost. We mark "VH" in row "cost" and column "machine 1"

	MC ①	MC ②	NCF ①	NCF ②	
Attributes/Machines	Machine 1	Machine 2	Machine 3	Machine 4	Machine 5
Cost	H	H	H	H	
Power	L	L	L	L	
Maximum spindle speed	H	H	M	M	
Maximum tool diameter	M	M	H	H	
Number of tools	M	M	—	—	
Cutting feed	M	M	M	M	
Traverse speed	M	M	M	M	
Positioning Precision (accuracy)	VH	VH	VH	VH	
Machine dimension	M	M	M	M	
Table area	M	M	M	M	
